# Nitrate of Strychnine in Alcoholism

**Published:** 1893-09

**Authors:** 


NITRATE OF STRYCHNINE IN ALCOHOLISM.—McConnell (*New York Medical Journal*, June 3, 1893) has used hypodermic injections one-twentieth of a grain of the above drug in twenty-five cases of alcoholism. He concludes: Simultaneously with the use of the remedy the craving for alcohol in inebriates diminishes, and in a few days is completely gone. There is a gradual restoration to physical and mental health, but as most of the cases treated relapsed in from one to eleven months the inhibiting power of the remedy is not permanent. While we have in strychnine a true antagonist to the action of alcohol, and one that will counteract its effects, the inebriate still requires aid which can scarcely be expected of drugs.—*Ex.*

